# Synthesis of flake-like graphene from nickel-coated polyacrylonitrile polymer

**DOI:** 10.1186/1556-276X-9-618

**Published:** 2014-11-18

**Authors:** Ho-je Kwon, Jun Mok Ha, Sung Ho Yoo, Ghafar Ali, Sung Oh Cho

**Affiliations:** 1 Department of Nuclear and Quantum Engineering, Korea Advanced Institute of Science and Technology, 291 Daehak-ro, Yuseong-gu, Daejeon 305-701, Republic of Korea; 2 Advanced Radiation Technology Institute, Korea Atomic Energy Research Institute, 29 Geumgu-gil, Jeongeup-si, Jeollabuk-do 580-185, Republic of Korea

**Keywords:** Polyacrylonitrile, Graphene, Pyrolysis, Graphene flake, Nickel

## Abstract

Graphene can be synthesized from polyacrylonitrile (PAN) polymer through pyrolysis. A metal catalyst such as nickel (Ni) is required for the conversion of the polymer to graphene. The metal catalysts can be placed either atop or underneath the polymer precursor. We observed that spatially non-uniform and disconnected graphene was fabricated when PAN film coated with a Ni layer was pyrolyzed, resulting in flake-like graphene. Formation of the flake-like graphene is attributed to the dewetting of the Ni layer coated on the PAN film. Dewetting phenomenon can be reduced by decreasing the pyrolysis temperature, and hence, more uniform graphene could be prepared. The effects of Ni coating thickness and the pyrolysis temperature on the fabricated graphene have been experimentally analyzed.

## Background

Ever since the discovery of graphene [1], research on graphene, a flat monolayer of carbon atoms arranged in a two-dimensional (2D) honeycomb lattice [2], has progressed rapidly. Due to the relatively simple and cheap procedures to obtain high-quality graphene [3] and its outstanding properties such as high electron mobility at room temperature [4], high intrinsic mechanical strength [5], high thermal conductivity [6], and complete impermeability to gas [7], graphene can be exploited in a variety of fields like electronics, photonics, energy generation and storage, sensor, and bio applications [3]. So far, many methods to obtain graphene have been developed, including mechanical cleavage of graphite [1], chemical exfoliation [8-10], epitaxial growth [11,12], chemical vapor deposition (CVD) [13-16], and solid-phase method [17-23].

The solid-phase method employs transition metals such as Ni and Cu as a catalyst to form graphene from solid-state carbon sources such as polymer, SiC, small molecule, and self-assembled monolayer. Particularly, in the method using polymer as a precursor of graphene, various polymers like polyacrylonitrile (PAN), polystyrene, and polymethylmethacrylate were used, and the polymer placed either atop or underneath metal catalysts were pyrolyzed in a reductive gas to form graphene [20-23]. When graphene is synthesized from a polymer precursor on a metal catalyst, an additional process to transfer the synthesized graphene on an insulator such as SiO_2_ is required for the application to electronic device [14,24]. This transfer procedure can result in the degradation of the synthesized graphene. The opposite case where polymer precursor is underneath a metal catalyst can solve this problem; however, few results have been reported on this case [22,23].

Here, we present systematic experimental results to synthesize graphene on a SiO_2_/Si substrate from PAN coated with a Ni film through pyrolysis. The Ni coating layer tends to be aggregated to form particulates due to dewetting [25] at a high pyrolysis temperature, and hence, the synthesized graphene was not generally continuous. The effects of the Ni film and pyrolysis temperature on the quality of graphene were investigated. As a consequence, continuously connected graphene could be prepared by reducing the pyrolysis temperature.

## Methods

Polyacrylonitrile (PAN, Sigma-Aldrich, St. Louis, MO, USA, *M*_w_ =150,000) (0.5 wt.%) dissolved in N,N-Dimethylformamide (DMF, Showa Chemical, Tokyo, Japan) was spin-coated on 1 × 1 cm^2^ SiO_2_ (300 nm thickness)/Si wafers. Subsequently, a Ni layer was coated on the spin-coated PAN/SiO_2_/Si substrates with a magnetron sputtering system. The sputtering rate was approximately 10 nm/min, and the thickness of the Ni layer was changed by the sputtering time. The Ni-coated PAN/SiO_2_/Si samples were pyrolyzed in a high-vacuum furnace; the vacuum level in the furnace was roughly 10^-5^ Torr.

During the pyrolysis process, the samples were gradually annealed with a heating rate of 8°C/min to a maximum temperature and then were quickly cooled down by moving the heating zone of the furnace to the opposite side. The maximum temperature was changed from 1,050°C to 700°C (Since we exploited a high-vacuum furnace made of quartz for pyrolysis, the maximum temperature has to be limited up to 1,100°C. Besides, although the melting point of nickel is approximately 1,450°C at 1 atm., a very thin nickel thickness (up to 200 nm) is easily agglomerated in the vacuum atmosphere. So the temperature of 1,050°C was selected as the maximum temperature. In the case of the minimum temperature range, the temperature where graphene is formed and the agglomeration of the nickel layer is suppressed was selected as the minimum temperature. Therefore, the temperature ranged from 1,050°C to 700°C was selected for the pyrolysis).

**Figure 1:**
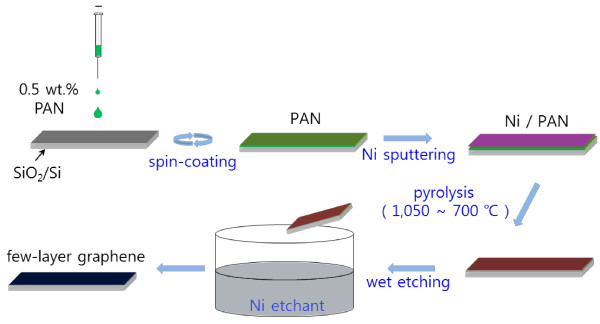
Schematic diagram to fabricate a multilayer graphene film from polyacrylonitrile.

After the pyrolysis, the coated Ni layer was removed with a Ni etchant (Nickel Etchant Type TFB purchased from TRANSENE, Danvers, MA, USA) at approximately 35°C for 2 to 4 h.The synthesized graphene on the substrate was characterized by Raman spectroscopy (532 nm laser, Ramboss500I, DongWoo Optron, Republic of Korea) and Raman mapping (514.5 nm laser, ARAMIS, Horiba Jobin Yvon, France). The surface morphologies of the products were observed using a field-emission scanning electron microscope (FE-SEM; JSM-7500 F, JEOL, Akishima, Japan) (Figure 1).

## Results and discussion

**Figure 2:**
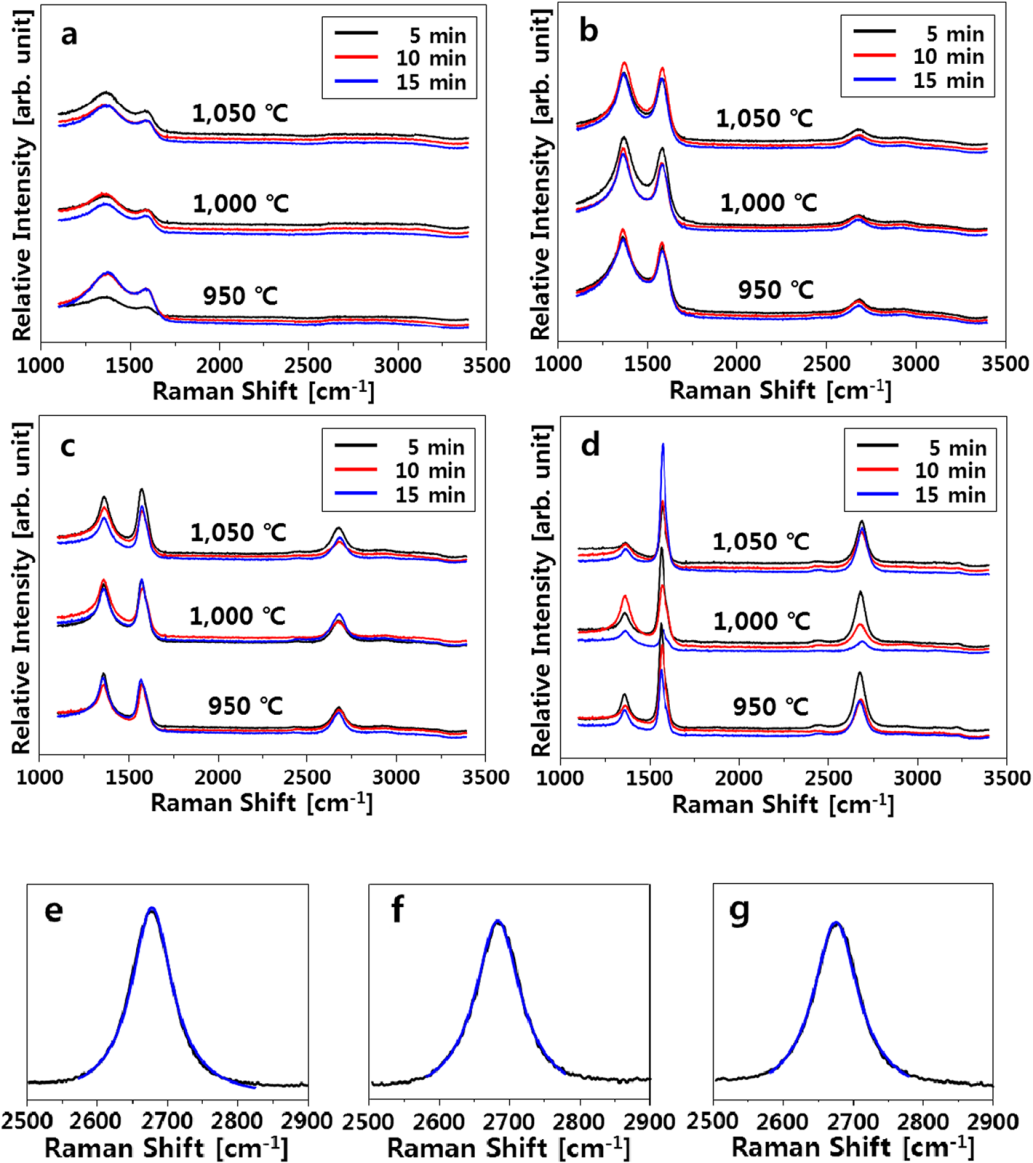
**Raman spectra of the samples at different thickness of the Ni coating.** The coated Ni thickness was 0 nm **(a)**, 20 nm **(b)**, 50 nm **(c)**, and 150 nm **(d)**. The Ni-coated PAN/SiO_2_/Si films were heated at different temperatures of 950°C, 1,000°C, and 1,050°C and holding time of 5, 10, and 15 min. Lorentzian fittings of the 2D peaks for the Ni(150 nm)/PAN/SiO_2_/Si films pyrolyzed at 950°C/5 min **(e)**, 950°C/10 min **(f)**, and 950°C/15 min **(g)** were shown. The black-colored lines are the original data, and the blue-colored lines denote the fitted data.

The effect of the coated Ni thickness on the synthesis of graphene was investigated. When the PAN film without a Ni coating was pyrolyzed, only two broad peaks appeared at approximately 1,360 cm^-1^ (D peak) and approximately 1,590 cm^-1^ (G peak) and no second-order Raman peak at 2,600 to 2,800 cm^-1^ corresponding to 2D peak of graphene was observed. This indicates that the PAN film was converted to amorphous carbon on the surface of the SiO_2_/Si substrate [26,27] (Figure 2a). However, when Ni film with the thickness of approximately 20 nm was coated on the PAN film, both D and G peaks became sharp and a small bump at approximately 2,700 cm^-1^ appeared (Figure 2b). As the coated Ni thickness was gradually increased from 20 to 150 nm, the shape of D, G, and 2D peaks became more distinct and the intensity ratio of D to G peaks (*I*_D_/*I*_G_) continuously decreased (Figure 2c,d). In addition, the Raman spectra were almost the same even though the maximum temperature was changed from 950°C to 1,050°C and the holding time at the maximum temperature was increased up to 15 min.

Full width at half maximum of the 2D peak (FWHM_2D_) of the Raman spectra shown in Figure 2d was approximately 70 cm^-1^, and the intensity ratio of G to 2D peaks (*I*_G_/*I*_2D_) was approximately 2 (Table 1). Moreover, it was confirmed that all the 2D peaks were fitted to a single Lorentzian profile (Figure 2e,f,g). Bi- and tri-layer graphene with a single Lorentzian profile of the 2D peak can be observed when the graphene was synthesized with CVD method and FWHM_2D_ of approximately 70 cm^-1^ and *I*_G_/*I*_2D_ ratio of approximately 2 are the characteristics of multilayer graphene [13]. In comparison, it was reported that turbostratic graphite has a single 2D peak while FWHM_2D_ is approximately 50 cm^-1^ and the position of 2D peak is upshifted by approximately 20 cm^-1^[28,29]. Consequently, we can speculate that the synthesized graphene from the Ni-coated PAN is a multilayer (3 ≤ number of layer) graphene.

**Table 1:** **Evaluation of FWHM**_**2D**_**, *I***_**(D)**_**/*I***_**(G)**_**, and *I***_**(G)**_**/*I***_**(2D) **_**for the Ni-coated PAN/SiO**_**2**_**/Si films after pyrolysis and subsequent nickel removal**

	**FWHM**_**2D **_**[cm**^**-1**^**]**	***I***_**(D)**_**/*I***_**(G)**_	***I***_**(G)**_**/*I***_**(2D)**_
Pyrolysis condition^a^			
1,050°C/5 min	66	0.358	1.33
1,050°C/10 min	72	0.357	1.92
1,050°C/15 min	66	0.169	2.94
1,000°C/5 min	66	0.308	0.97
1,000°C/10 min	79	0.819	2.76
1,000°C/15 min	67	1.000	2.11
950°C/5 min	67	0.338	1.86
950°C/10 min	73	0.300	2.62
950°C/15 min	71	0.392	1.88
Average	70.77	0.486	2.03

^a^Ni thickness is 150 nm.

**Figure 3:**
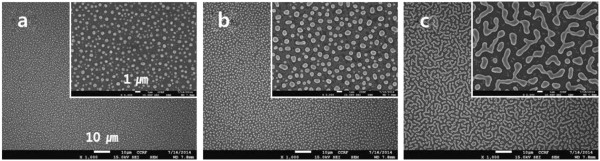
**FE-SEM images of the Ni-coated PAN films after the pyrolysis at 950°C/10 min.** Where the coated Ni thickness is 40 nm **(a)**, 100 nm **(b)**, and 200 nm **(c)**, respectively.

The evolution of the surface morphology at different Ni thickness was investigated. As can be seen in Figure 3, an agglomerate of micrometer- or nanometer-sized Ni particles were observed after the pyrolysis at 950°C. Formation of the Ni particles from the Ni film is attributed to dewetting phenomenon [25]. The size of the produced Ni particles increased with increasing the thickness of the Ni coating (Figure 3a,b,c).

**Figure 4:**
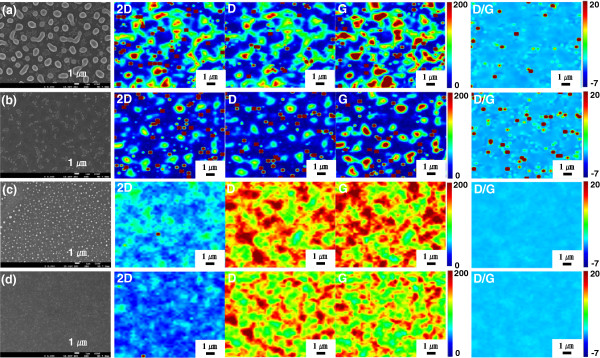
**Raman mapping images obtained before and after Ni etching for Ni/PAN/SiO**_**2**_**/Si films pyrolyzed at 1,050°C/15 min.** The coated Ni thickness is 150 nm **(a, b)** and 20 nm **(c, d)**, respectively. The FE-SEM images on the very left hand side of each image set are arbitrarily selected images for the reference and do not correspond to the Raman mapping images (mapping area, 15 × 15 μm^2^; scale bar, 1.0 μm).

**Figure 5:**
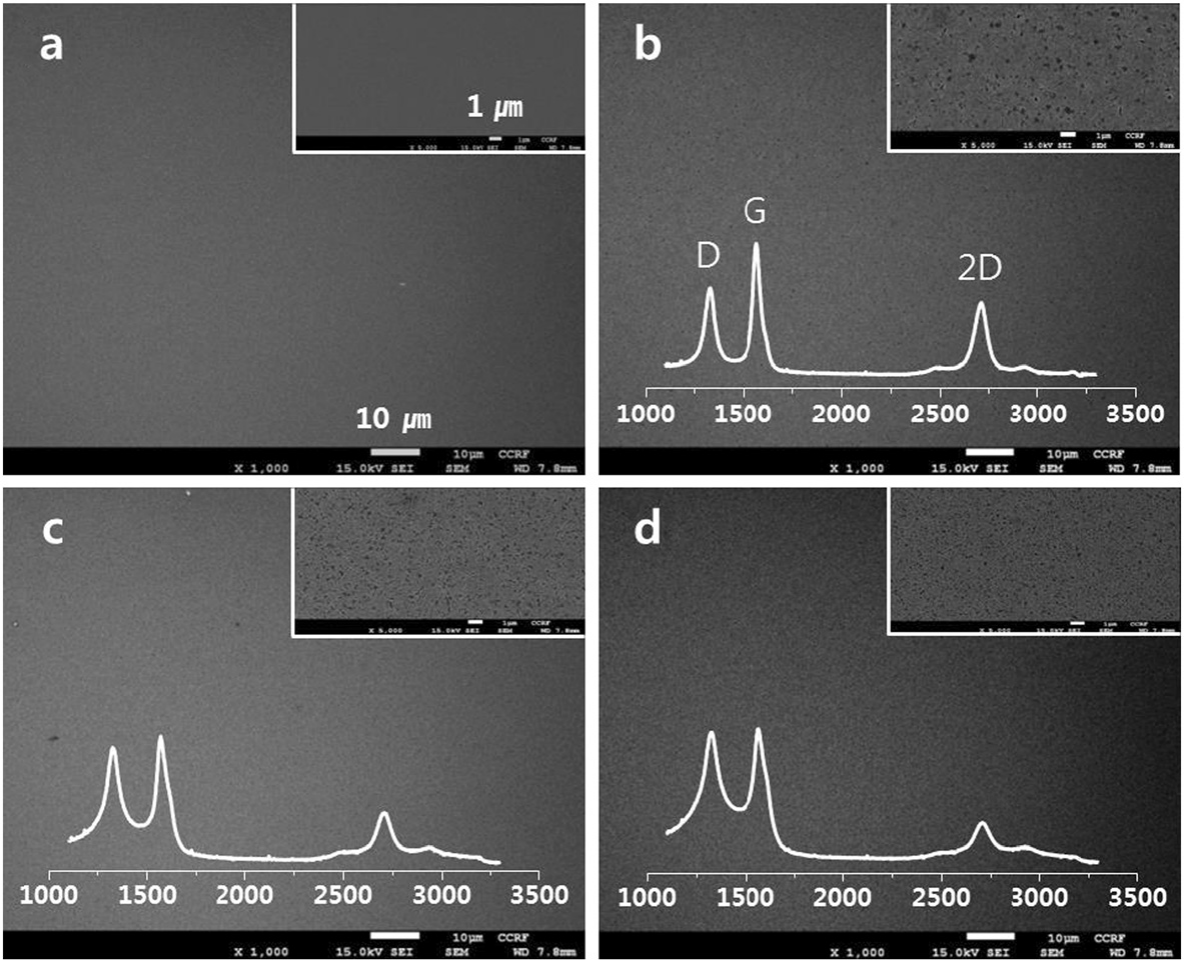
**FE-SEM images of the Ni layers on PAN/SiO**_**2**_**/Si.** After the pyrolysis at 800°C/10 min **(b)**, 750°C/10 min **(c)**, and 700°C/10 min **(d)**. FE-SEM image of a pristine film is also shown in **(a)**. Their corresponding Raman spectra of the samples after Ni etching were included in the images.

**Figure 6:**
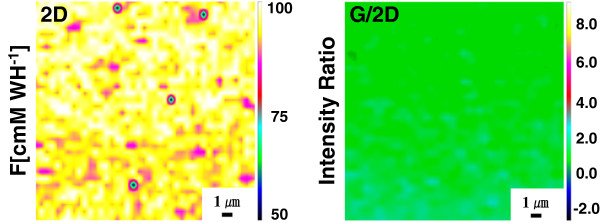
**Raman mapping images obtained after Ni removal for the Ni/PAN/SiO**_**2**_**/Si film pyrolyzed at 750°C for 10 min.** Mapping area, 15 × 15 μm^2^; scale bar, 1 μm.

In order to identify that graphene is formed from PAN even though dewetting of the catalytic Ni film occurred, Raman mapping analysis on the pyrolyzed Ni-coated PAN films was carried out (Figure 4). The first column in Figure 4 shows the FE-SEM images of the Ni-coated PAN films with different Ni coating thickness before and after Ni etching. The other four columns denote 2D, D, G, and D/G mapping images, respectively. The blue color in the Raman mapping images indicates the lowest intensity of the signal, whereas brown color indicates the highest intensity. It is clearly shown that graphene was sparsely formed on the surface of SiO_2_, which is caused by the dewetting of Ni catalyst film. Moreover, from the FE-SEM images (the first column of Figure 4a,c) and 2D Raman mapping images (the second column of Figure 4a,c) of the pyrolyzed Ni-coated PAN films before Ni removal, we can find that graphene was mainly formed both on and around the Ni particles. The presence of graphene both on and around the Ni particles can be explained by the solubility of carbon source on the catalytic Ni particles and the diffusion of carbon from the Ni particles to outside of the particles during the pyrolysis procedure [30,31]. In addition, the FE-SEM images (the first column of Figure 4b,d) and 2D Raman mapping images (the second column of Figure 4b,d) of the films after Ni removal show that graphene was primarily synthesized underneath the Ni particles. Therefore, very non-uniform, flake-like graphene was fabricated by the pyrolysis of the Ni-coated PAN films due to the dewetting of the catalytic Ni films during the pyrolysis process.Flake-like graphene might be useful for certain applications, but uniform and interconnected graphene is necessary for the applications to electronic devices. Since such flake-like graphene is produced because of dewetting of Ni film on PAN, suppressing of dewetting is required for the fabrication of a continuous graphene film. As shown in Figure 3, when the thickness of Ni film on PAN was increased, the dewetting phenomenon was alleviated. However, a too thick Ni film may lead to the difficulty in removing the Ni film after the pyrolysis. Alternatively, we tried to decrease the pyrolysis temperature. When the pyrolysis temperature was decreased from 1,050°C to 800°C (Figure 5b), dewetting was significantly suppressed. However, many pores were still observed on the pyrolyzed nickel layer. Both the density and the size of the pores were gradually decreased as the pyrolysis temperature was further decreased down to 700°C, and consequently, a more uniform Ni film with less defects was prepared (Figure 5d). Definitely, lower pyrolysis temperature could almost completely prevent the dewetting of Ni coating. However, the quality of the synthesized graphene was deteriorated with the decrease in the pyrolysis temperature. As can be seen in the Raman spectra of Figure 5b,c,d, the intensity of D peak was gradually increased while the intensity of 2D peak was gradually decreased as the pyrolysis temperature was decreased, indicating that graphene with lower quality is produced.After eliminating the Ni layer, Raman mapping of the graphene film prepared by the pyrolysis process at 750°C was carried out to evaluate the spatial uniformity and coverage of the synthesized graphene (Figure 6). Although a slight deviation exists in the 2D map, the intensity ratio of G to 2D exhibits a considerably identical color tone, which means that the synthesized graphene is fairly uniform.

## Conclusions

We have demonstrated that spatially non-uniform and flake-like graphene is synthesized when Ni-coated PAN film is pyrolyzed at a high temperature. Such non-uniform graphene is produced due to the dewetting of the Ni layer coated on the PAN film. Dewetting phenomenon can be reduced by increasing the Ni thickness and/or by decreasing the pyrolysis temperature. However, as the pyrolysis temperature is decreased, graphene with lower quality is synthesized. Therefore, it is important to optimize both the Ni thickness and the pyrolysis temperature considering the necessary quality of the synthesized graphene and required spatial uniformity for certain applications. In addition, non-uniform and flake-like graphene is not so good for the application to electronic devices; however, such flake-like graphene might be useful for certain applications of graphene (e.g., gas sensor and energy storage [32,33]) if the flake size can be controlled through future studies.

## Competing interests

The authors declare that they have no competing interests.

## Authors’ contributions

HK carried out the experiments, contributed to the interpretation of the data and drafted the manuscript. JMH contribute to measure Raman spectrum. SHY and GA assisted in the experiments. SOC supervised the whole study. All authors read and approved the final manuscript.
